# Deep neck infection complicating lymphadenitis caused by *Streptococcus intermedius *in an immunocompetent child

**DOI:** 10.1186/1471-2334-6-61

**Published:** 2006-03-22

**Authors:** Donato Rigante, Teresa Spanu, Lorenzo Nanni, Assunta Tornesello, Maurizio Sanguinetti, Tiziana D'Inzeo, Achille Stabile, Giovanni Fadda

**Affiliations:** 1 Department of Pediatric Sciences, Università Cattolica Sacro Cuore, Rome, Italy; 2 Institute of Microbiology, Università Cattolica Sacro Cuore, Rome, Italy; 3 Division of Pediatric Surgery, Università Cattolica Sacro Cuore, Rome, Italy

## Abstract

**Background:**

*Streptococcus intermedius *belongs to the *Streptococcus anginosus *group. It is part of the normal flora of the human mouth, but it can be etiologically associated with deep-site infections.

**Case presentation:**

We present a case of deep neck infection complicating *Streptococcus intermedius *lymphadenitis, which developed in an immunocompetent 14-year-old boy with a history of recent dental work. The infection was ultimately eradicated by a combined medical and surgical approach.

**Conclusion:**

Our report suggests that combined medical and surgical therapy is essential for the complete resolution of deep infections caused by *Streptococcus intermedius*. Molecular biological techniques can be useful in guiding the diagnostic investigation and providing insight into the possibility of occult abscesses, which are particularly common with *Streptococcus intermedius *infections.

## Background

*Streptococcus intermedius *belongs to the *Streptococcus anginosus *group (SAG) and is specifically associated with the formation of abscesses, which can also develop at a distance as a result of hematogenous spread [1]. The complete clinical spectrum of infections caused by this group of bacteria has yet to be defined. Here we report a case of *S. intermedius *infection of the lateral cervical lymph-nodes, which was complicated by deep neck infection.

## Case presentation

A 14-year old boy presented to his local physician with a 2-week history of marked swelling in the left lateral cervical region. There was no history of fever, headache, rash, or joint pain. One month earlier the child had completed a course of treatment with amoxicillin-clavulanate for pharyngotonsillitis. This infection had followed on the heels of a case of left otitis media, which had been successfully treated with the same antibiotic. Over the past three months, he had undergone repeated orthodontic procedures which were associated with some degree of gingivitis. The parents could not recall whether or not any treatment had been prescribed for the gingival lesions.

After five days of treatment with ceftriaxone (25 mg/kg once daily) the cervical mass was unchanged and the child was referred to our hospital. On admission, he was found to be in good clinical condition and all vital signs were normal, including temperature. The physical examination revealed a firm non-tender mass on the left side of the neck. It was relatively non-elastic in consistency and the overlying skin was mildly erythematous. The palatine tonsils were both hypertrophic and the left one was medially displaced; velar and tongue motility were normal. The rest of the physical examination was unremarkable. Laboratory data included a peripheral white blood cell count of 10.3 × 10^9^/liter with 42% segmented neutrophils, hemoglobin 12.9 g/dl, platelet count 202 × 10^9^/liter, erythrocyte sedimentation rate (ESR) 68 mm/h, C-reactive protein (CRP) 44.1 mg/liter, and ferritin 249 ng/ml. The Mantoux intradermal reaction was negative at 72 hours. Blood cultures were persistently negative, and angiotensin-converting enzyme levels, serum copper levels, and neutrophil chemiluminescence findings were within normal ranges. Serological tests for Epstein-Barr virus, *Bartonella henselae*, Cytomegalovirus, and *Toxoplasma gondii *were all negative. Ultrasound of the left lateral cervical region showed a solid mass measuring 3 × 3.5 cm around the carotid bifurcation. It was surrounded by small lymph nodes with a maximum diameter of 1.5 cm. The chest X-ray and abdominal ultrasound findings were normal.

On the suspicion that the child was suffering from mycobacteriosis provoked by the recent orthodontic work, empirical treatment with oral clarithromycin (250 mg BID) and ciprofloxacin (250 mg BID) was started. After seven days, an open lymph-node biopsy revealed purulent drainage, which was collected for histopathologic and microbiological analyses. Two drains were positioned within the wound and attached to low-grade suction for ten days. The cytologic findings were consistent with lymphadenitis. In light of the microbiological findings (discussed below), clarithromycin was discontinued, and the patient was placed on cefotaxime (1000 mg three times/day). One week later, the dimensions of the neck mass seemed to be slightly decreased on ultrasonography. However, a computerized-tomography (CT) scan of the head, neck, and mediastinum showed that the swelling was much more extensive than previously thought. It was only partially lymphoadenopathic, and fluid-containing areas alternating with zones of non-uniform enhancement were observed throughout the left parotid region, parapharyngeal space, tonsil pillar, the posterolateral wall of the pharynx, the posterior cervical and carotid spaces, the sternocleidomastoid muscle, and the superficial musculofascial layer. After two more weeks of treatment, an objective improvement was noted. Cefotaxime was discontinued and the patient was discharged on acetoxyethylcefuroxime (500 mg BID). One month later, the child returned to our hospital for a follow-up visit after completion of four weeks of acetoxyethylcefuroxime therapy. An area of persistent painless swelling was noted and confirmed by CT in the left submandibular region. Surgical exploration revealed a large abscess in the submandibular salivary gland. After vessel and duct resection, the decision was made to proceed with the complete removal of the infected gland. After a total of six weeks of therapy, acetoxyethylcefuroxime was discontinued, and patient's neck CT scan confirmed the complete resolution of the abscess. He was discharged after a total hospitalization of 32 days and is currently being followed in our outpatient clinic. Twelve months after discharge, there are no signs of abscess recurrence or other types of infection.

## Microbiological diagnosis

Direct Gram stain of the node drainage revealed a few Gram-positive cocci; Ziehl-Nielsen stain detected no acid-alcohol resistant bacteria. After a few days of incubation at 37°C in air with 5% CO_2_, pus cultures on 5% sheep blood agar (bioMérieux, Marcy-L'Etoile, France) produced non-beta-hemolytic colonies of Gram-positive cocci, which were presumptively identified by the API 20 Strep System (bioMérieux) as a member of the SAG. *In vitro *antimicrobial susceptibility testing performed using the microdilution method with lysed-horse blood-supplemented Mueller-Hinton broth [2] indicated that the isolate was susceptible to penicillin (MIC ≤ 0.12 mg/L); ampicillin (MIC ≤ 0.25 mg/L); erythromycin (MIC ≤ 0.06 mg/L); clindamycin (MIC ≤ 0.06 mg/L); and vancomycin (MIC ≤ 0.5 mg/L). Cultures for mycobacteria yielded no growth.

Cultures of the purulent material deriving from the salivary-gland abscess yielded the same organism isolated from the initial lymph-node biopsy. The two isolates were thus subjected to more extensive characterization. Phenotypic identification based on Whiley's scheme [3] revealed a biochemical profile consistent with *S. intermedius*, confirmed by the results of 16S rRNA bacterial sequencing through eubacterial primers with the procedure described by Clarridge et al. [4,5]. The presence of the intermedilysin (*ily*) gene was also confirmed by means of a PCR assay, which has been previously described [6].

## Discussion

*Streptococcus intermedius*, *S. anginosus*, and *S. constellatus*, the three members of the SAG, are generally considered to be normal inhabitants of the human oral cavity [5]. They are also known to be the cause of endogenous infections not only in the oral cavity, but also at deep sites [7]. *S. intermedius *is of particular interest since it shows tropism for the brain and liver. Infection with this species is typically linked to abscess formation [4,5,7,8], which is often deep-seated and/or associated with hematogenous spread.

It is not easy to differentiate *S. intermedius *from the other two SAG species with routine identification procedures [9,10]. In most clinical laboratories, species-level identification of SAG isolates cannot be achieved because of the low discriminatory power of automated commercial identification systems. The identification of the SAG species based on molecular biological techniques can be useful in guiding the diagnostic investigation and providing insight into the possible role of coinfecting organisms and the probability of occult abscesses, which are more likely with *S. intermedius *infections [5,6,10,11]. The SAG species also differ in terms of the virulence factors they produce. For example, the enzymes α-*N*-acetylneuramidase (sialidase) and hyaluronidase, which destroy host tissues and presumably convert them into small nutrients to be utilized in bacterial growth [6], are both known to be produced by *S. intermedius*, whereas *S. constellatus *produces only hyaluronidase, and *S. anginosus *produces neither. Strains of *S. intermedius *also secrete a human-specific cytolysin known as intermedilysin, and its expression has recently been correlated with strain pathogenicity or with the severity of *S. intermedius *infections [6]. To detect the *ily *gene we used the primers and protocol described in 2000 by Nagamune *et al*. [6]. The same investigators later reported cases of non-specific amplification with their protocol [10] and they have since then developed an improved *ily *gene-specific PCR primer set, which specifically amplifies the *ily *gene and the 3'-flanking region in *S. intermedius*, but not in other members of the SAG group. We did not experience any non-specific amplification with the original primer set, but use of the newer protocol is strongly recommended for accurate detection of *S. intermedius*, which is undoubtedly the most pathogenic of the three SAG species.

Although deep neck infections are increasingly less frequent than they were in the past, they are still associated with significant morbidity and mortality rates as high as 40–50% [12]. Improvements in antimicrobial therapy and dental care have played a significant role in decreasing the frequency of these infections. In the pre-antibiotic era most deep neck infections were complications of pharyngeal infections. The most common abscess sites were the lateral pharyngeal space, followed by the submandibular, Ludwig's, and retropharyngeal spaces [13]. Dental infection leading to cervical lymphadenitis and subsequent abscess formation seems to be actually the most common cause of deep infections located in the lateral pharyngeal space [13]. Deep neck abscesses are also associated with diabetes mellitus, immunocompromised states, including HIV infection and AIDS, poor dental health, and previous invasive medical procedures [12-14]. Cases have been reported in immunocompetent patients and life-threatening infections can even occur in previously healthy individuals [13,14].

Our patient was immunocompetent with no underlying chronic disease and his medical history was unremarkable except for previous pharyngotonsillitis and repeated dental procedures in the foregoing months. The initial presentation was that of cervical lymphadenitis, but the infection subsequently extended to involve other spaces. The infection was initially misdiagnosed as mycobacteriosis. Its failure to respond to empirical antibiotic therapy led to a lymph-node biopsy and both pathologic report and pus cultures confirmed the presence of a neck abscess caused by *S. intermedius*.

Deep neck infections are often polymicrobial [13]: the predominant species involved are viridans streptococci, followed by *Staphylococcus epidermidis *and *Staphylococcus aureus*, but anaerobes are also common [13]. In the case reported here, the only pathogen identified was *S. intermedius*. This species is usually found to be susceptible to penicillins and cephalosporins [15,16], but successful treatment of deep neck infections caused by *S. intermedius *must also include surgical approaches [12,13]. In our patient the initial therapy with clarithromycin and ciprofloxacin, which was empirically prescribed on the assumption that he was suffering from a mycobacterial infection, was later replaced with cefotaxime, administered for three weeks after the isolation of *S. intermedius*. In the end, the infection was eradicated by surgical removal of the infected submandibular salivary gland and a six-week cycle of acetoxyethylcefuroxime.

## Conclusion

The report describes a case of cervical lymphadenitis complicated by severe deep neck infection caused by *S. intermedius *in a fully immunocompetent adolescent boy with a history of pharyngotonsillitis and recent orthodontic work. The initial presentation of these infections can be fairly non-specific and precious time can be lost while more common causes of lymphadenopathy are being excluded. Distinguishing *S. intermedius *from other SAG species is important because it carries a substantial risk for persistent or recurrent abscesses. Molecular techniques allow rapid and accurate detection of *S. intermedius *and thus represent a valuable tool for the management of severe infections caused by this pathogen. Our findings suggest that *S. intermedius *should be suspected in all cases of deep neck infections in which the patient has recently undergone dental work. Pediatricians should not underestimate the importance of the isolation of this species from clinical specimens.

## Competing interests

The author(s) declare that they have no competing interests.

## Authors' contributions

DR and TS followed the patient and drafted the manuscript. MS, TDI and GF carried out the laboratory studies of the patient. Surgery was performed by LN. AT and AS provided clinical details of the case. All authors read and approved the final manuscript.

## Pre-publication history

The pre-publication history for this paper can be accessed here:


